# Catch rate composition affects assessment of protected area impacts

**DOI:** 10.1038/s41467-021-21607-4

**Published:** 2021-03-11

**Authors:** Jonathan R. Sweeney

**Affiliations:** National Marine Fisheries Service, Pacific Islands Fisheries Science Center, National Oceanic and Atmospheric Administration, Honolulu, HI USA

**Keywords:** Conservation biology, Environmental economics, Economics

**Arising from** Lynham et al. *Nature Communications* 10.1038/s41467-020-14588-3 (2020)

Lynham et al.^1^ assess the impact of marine national monument expansion on catch rates in Hawaii’s longline fishery, finding little, if any negative impact. Another paper, recently published by Chan^2^, also assesses the impact of Papahānaumokuākea Marine National Monument (PMNM) expansion, but finds, for vessels with a history of fishing in PMNM, expansion decreased catch rates by 7% and revenues by 9%. I examine the source of this discrepancy and find catch rate composition to critically affect the underlying trends in data with which models are fit and are likely the source of the conflicting findings. This analysis suggests that aggregate commercial catch rate is a more robust measure of catch per unit effort (CPUE) for Hawaii’s deep-set longline fishery, and I recommend a reanalysis of Lynham et al.’s^1^ model using this measure.

Using the same National Oceanic and Atmospheric Administration Observer Program data source as Lynham et al.^1^, I reconstructed the time series of catch-per-1000-hooks used to assess fishery impacts in their paper, combining only bigeye and yellowfin tuna catch. I then built a second measure of catch-per-1000-hooks from the same data source combining 11 commercial species frequently caught in Hawaii’s deep-set longline fishery. The two measures show opposite changes in catch rate in the year following PMNM expansion, with aggregate commercial CPUE declining and Bigeye + Yellowfin CPUE increasing (Fig. 1a, dashed lines). The decline in aggregate commercial CPUE corresponds to the declines documented by Chan^2^, who also used a measure of aggregate commercial catch rate and revenue. It is also noted that the increase in Bigeye + Yellowfin CPUE documented by Lynham et al.^1^ in 2017 quickly dissipates when data from 2018 and 2019 were included, whereas aggregate commercial CPUE continues to decline in those years. To compare the two measures of catch composition in economic terms, I approximated the average revenue per unit effort (RPUE) for aggregate commercial and Bigeye + Yellowfin (Fig. 1b). Both indices of RPUE increase the year following PMNM expansion and then decline when data from 2018 and 2019 were included. The consistent differences between short and long period averages suggest that factors beyond monument expansion have a large influence on CPUE and RPUE trends.

**Fig. 1:**
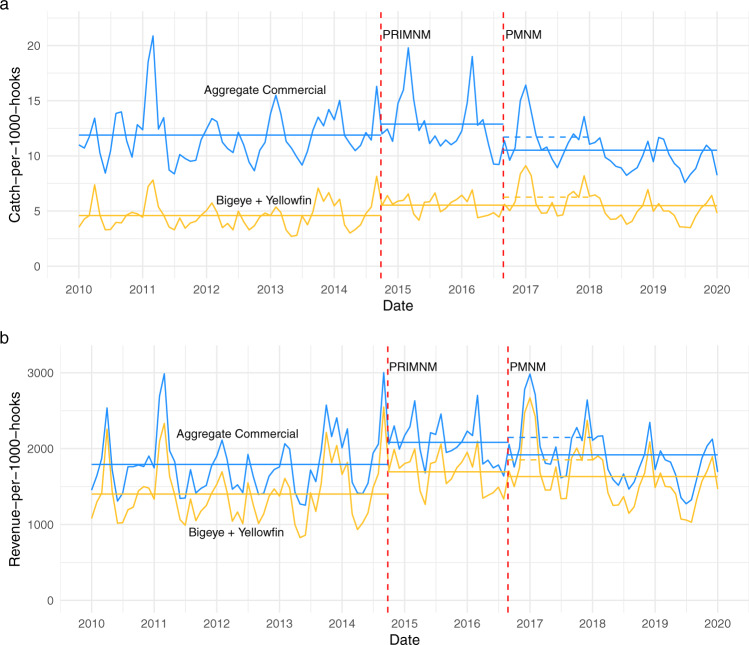
Monthly CPUE and RPUE calculated using two measures of catch composition. **a** Catch-per-1000-hooks measured by aggregate commercial catch (blue) and Bigeye + Yellowfin tuna catch (gold) calculated using data from the NOAA Observer Program. Solid horizontal lines indicate average catch rates over the designated time periods. Dashed lines indicate average catch rates in the post-PMNM expansion period presented in Lynham et al.^1^. The two red dashed lines indicate the Pacific Remote Island Marine National Monument expansion (PRIMNM) and the Papahānaumokuākea Marine National Monument expansion (PMNM). **b** Revenue-per-1000-hooks aggregated for 11 commercially caught species (blue) and Bigeye +  Yellowfin (gold). Revenue-per-1000-hooks was approximated by multiplying the number of individuals caught by the average value of individual fish for each species, then dividing by effort measured in 1000 s of hooks. The average value of individual fish was calculated using observed fish sales from 2010 to 2019 from Hawaii’s dealer data. The two red dashed lines indicate the Pacific Remote Island Marine National Monument expansion (PRIMNM) and the Papahānaumokuākea Marine National Monument expansion (PMNM).

To understand what drives the differences between aggregate commercial and Bigeye + Yellowfin CPUE trends, I constructed separate measures of catch-per-1000-hooks for each of the 11 commercial species (Fig. 2). The primary feature that stands out is that yellowfin is the only species to show a substantial increase in catch rate in the year after PMNM expansion. All other species show a decline or little change. Constructing a metric that only combines bigeye and yellowfin tuna gives extra weight to an anomalous increase in yellowfin catch. A second relevant feature from this analysis is that yellowfin tuna, when averaged from 2010 to 2019, has only the fourth highest catch rate in the fishery, with bigeye tuna, mahimahi, and sickle pomfret ranked as the top three, respectively. Given these features of the data, aggregate commercial catch rate should be the preferred measure of CPUE for assessing impacts to Hawaii’s longline fishery. It is robust to single-species anomalies and better accounts for the respective contribution of each commercially caught species to overall landings.

**Fig. 2:**
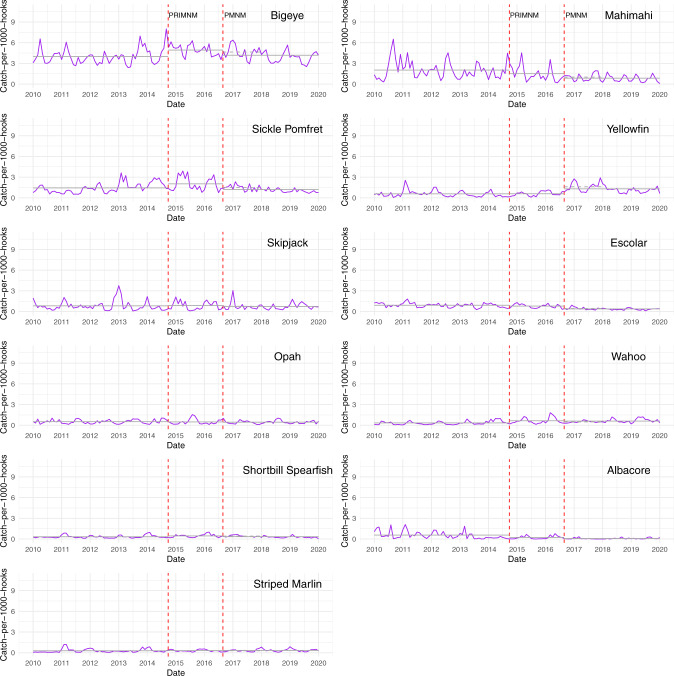
Monthly CPUE for 11 commercial species caught in Hawaii’s deep-set longline fishery. Catch-per-1000-hooks for 11 commercially caught species in Hawaii’s deep-set longline fishery calculated using data from the NOAA Observer Program. Solid grey lines indicate average catch rates over the designated time periods. Dashed grey lines indicate average catch rates in the post-PMNM expansion period presented in Lynham et al.^1^. The two red dashed lines indicate the Pacific Remote Island Marine National Monument expansion (PRIMNM) and the Papahānaumokuākea Marine National Monument expansion (PMNM).

Although society-wide benefits of protected areas may still exceed locally concentrated costs, it is critical to accurately assess the economic damages borne by those most affected by government regulations. This analysis suggests that aggregate commercial catch rates provide a more accurate assessment of economic impacts to Hawaii’s longline fishery and appear to indicate that Lynham et al.’s^1^ findings may be, in part, derived from using a limited catch rate measure that has a positive trend bias. It is also clear from the analysis that, in addition to understanding the impacts of protected area expansion, attention should be directed toward understanding the causes of shifting catch composition, which underlie those impacts, and suggests that pelagic ecosystems are changing.

## Reporting summary

Further information on research design is available in the Nature Research Reporting Summary linked to this article.

## Supplementary information

Reporting Summary

## Data Availability

The Observer Program data [https://www.fisheries.noaa.gov/inport/item/16865] and Hawaii’s Dealer data [https://www.fisheries.noaa.gov/inport/item/5610] analyzed in the study are not publicly available due to containing business confidential information and a non-disclosure agreement signed by J.R.S.

## Code availability

The code used to summarize the data and generate the figures in this study is publicly available in the following GitHub repository: https://github.com/JonathanSweeney-NOAA/PMNM-matter-arising.
